# Sensitivity of the Goldfish Motion Detection System Revealed by Incoherent Random Dot Stimuli: Comparison of Behavioural and Neuronal Data

**DOI:** 10.1371/journal.pone.0009461

**Published:** 2010-03-01

**Authors:** Olivia Andrea Masseck, Sascha Förster, Klaus-Peter Hoffmann

**Affiliations:** Department of General Zoology and Neurobiology, Ruhr University Bochum, Bochum, Germany; University of New South Wales, Australia

## Abstract

**Background:**

Global motion detection is one of the most important abilities in the animal kingdom to navigate through a 3-dimensional environment. In the visual system of teleost fish direction-selective neurons in the pretectal area (APT) are most important for global motion detection. As in all other vertebrates these neurons are involved in the control of slow phase eye movements during gaze stabilization. In contrast to mammals cortical pathways that might influence motion detection abilities of the optokinetic system are missing in teleost fish.

**Results:**

To test global motion detection in goldfish we first measured the coherence threshold of random dot patterns to elicit horizontal slow phase eye movements. In addition, the coherence threshold of the optomotor response was determined by the same random dot patterns. In a second approach the coherence threshold to elicit a direction selective response in neurons of the APT was assessed from a neurometric function. Behavioural thresholds and neuronal thresholds to elicit slow phase eye movements were very similar, and ranged between 10% and 20% coherence. In contrast to these low thresholds for the optokinetic reaction and APT neurons the optomotor response could only be elicited by random dot patterns with coherences above 40%.

**Conclusion:**

Our findings suggest a high sensitivity for global motion in the goldfish optokinetic system. Comparison of neuronal and behavioural thresholds implies a nearly one-to-one transformation of visual neuron performance to the visuo-motor output. In addition, we assume that the optomotor response is not mediated by the optokinetic system, but instead by other motion detection systems with higher coherence thresholds.

## Introduction

The ability of the visual system to detect global motion is essential for almost all animals [1]. By analyzing global motion one's own locomotor velocity, position in space, and distances to objects can be estimated [2], [3], [4], [5]. To assess the ability of the visual system to perceive global motion, random dot patterns with different coherences have become a proven tool in neuroscience [6], [7]. In random dot patterns with low coherence global motion cannot be extracted on the basis of individual dots. Instead global motion integration has to take place to detect the direction and speed of the stimulus. The capability to perceive global motion is quite different in various species. Humans and monkeys are able to recognise global motion down to only 5% coherent motion in a random dot pattern, i.e. 5% of the dots move in one direction and the remaining 95% move randomly [7], [8]. Ferrets and pigeons are worse in detecting global motion and reach thresholds of 30% and 44%, respectively [7], [9].

To perceive global motion local motion signals have to be integrated over space and time. In mammals local motion detectors like orientation and direction selective neurons in V1 are only capable to encode motion signals in spatially distinct areas due to their limited receptive field sizes [10]. Higher brain areas have to integrate these local motion signals to extract global motion. In primates the middle temporal visual area (MT) which receives preprocessed information from V1 is known to encode global motion information from a given stimulus [6]. Parallel to the perception of motion activity from direction selective neurons in MT and medial superior temporal area (MST) drive smooth pursuit and optokinetic reactions (OKR) via corticofugal projections to the pontine nuclei and the nucleus of the optic tract (NOT) [11]. A behavioural consequence of these pathways is to stabilize an image of an object or the whole visual scene on the retina. Smooth pursuit keeps a moving object of interest on the fovea. The OKR describes the kind of eye movement, present in all vertebrates, that stabilizes the whole image on the retina during own and environmental movements. Image stabilization is achieved by moving the eyes within the same direction and with the same velocity as the occurring retinal image slip. The OKR is not the only reflex which supports gaze stabilization, also the optomotor response (OMR) pervades this problem. During OMR the retinal image is stabilized when an animal swims or runs in the same direction and with the same speed as the occurring optic flow. Fish typically use the OMR to maintain their position in flowing water. Measuring OMR and OKR has become popular to determine the genetics of these behaviours in zebra fish [12], [13]. But little is known about the neuronal substrate of the OMR and obtained results were discussed controversially: Springer et al. [14] showed that the OMR of goldfish depends upon an intact tectum opticum: after an ablation of both tectal lobes the OMR of goldfish was completely abolished. In contrast, bilateral laser ablation of the zebrafish opticum tectum did not alter the OMR [15]. This at the first glance contradiction might be explained by the extent of the lesions: In the latter case only retinorecipient layers of the opticum tectum were ablated, whereas deeper layers, where premotor functions are located, were left intact.

The neuronal substrate for the optokinetic response is however well investigated in a variety of vertebrate species. In mammals direction-selective neurons in the pretectal NOT and the accessory optic system (AOS), composed of the dorsal terminal nucleus (DTN), the lateral terminal nucleus (LTN) and the medial terminal nucleus (MTN) in mammals, are required for this behaviour [16], [17], [18], [19], [20], [21]. Each nucleus receives direct retinal input and contains direction-selective neurons with large area receptive fields encoding a specific retinal slip direction, e.g.: NOT and DTN neurons code for horizontal ipsiversive retinal slip.

In tetrapods other than mammals gaze stabilization is mediated by direction-selective neurons in the pretectal nucleus lentiformis mesencephali (nLM) and the nucleus of the basal optic root (nBOR), though only the nBOR is considered as part of the AOS [22]. Again both structures receive direct retinal input.

In teleost fish slow phase eye movements for gaze stabilization are mediated by direction selective neurons in the pretectal area (APT) [23], [24], and in the case of chondrichtyans in the corpus geniculatum laterale (Cgl) [25], both areas are supposed to be homologous to the accessory optic system (AOS) and the NOT/nLM of tetrapods. In contrast to tetrapods neurons in the APT and Cgl are sensitive to the whole range of directions of retinal slip and a segregation of preferred directions into different nuclei has not yet occurred [23], [24], [25]. In addition, visual direction-selective neurons can also be found in the tectum opticum of fish [26]. Admittedly, these neurons are not involved in the execution of slow phase eye movements, but rather in the control of orienting, locomotion and posture [27]. In fish connections between neurons in the tectum opticum and direction selective neurons in the pretectum, have not yet been described and both systems seem may operate independently from each other.

So far the sensitivities of the optokinetic response and the optomotor response as well as their neuronal substrates for global motion detection are not described. Therefore in this study we applied the well established method of varying the coherence level of moving random dot patterns to determine and compare the thresholds for OKR and OMR as well as the neurometric function of neurons in the APT in goldfish. The data are discussed to answer the question whether OKR and OMR are served by the same or different neuronal populations.

### Objectives

In a first step we measured the OKR in a behavioural paradigm during stimulation with random dot stimuli of different coherence levels to ascertain the threshold of the optokinetic system for global motion detection. In a second step visual direction-selective neurons in the APT, mediating the OKR in teleost fishes, were examined with the same motion stimuli to understand the transformation of sensory inputs to corresponding motor outputs.

At last we measured the optomotor response to stimuli with different coherence levels and determined its threshold. If different thresholds for the OKR and OMR exist, this would provide evidence for different underlying circuitries in mediating the OKR and OMR.

Our study shows high global motion detection abilities of the goldfish optokinetic system in comparison to other species. And a significant higher threshold for eliciting the OMR proposes that the APT of teleost fish is probably not involved in the execution of the OMR.

## Materials and Methods

Data from 19 goldfish were included in the present study. Animal size varied between 5 cm–15 cm in length and included animals of both sexes. All experiments were approved by the local authorities (Regierungspräsidium Arnsberg) and carried out in accordance with the Deutsche Tierschutzgesetz of 12 April 2001, the European Communities Council Directive of 24 November 1986 (S6 609 EEC) and NIH guidelines for care and use of animals for experimental procedures.

### OKR Stimuli

For visual horizontal wholefield stimulation different videos projected by a beamer, ranging from 0% coherence up to 100% coherence in 10% steps were used. All videos were custom made in MATLAB (7.01). Here 100% coherence means that all dots moved into one direction, whereas e.g. in a 70% coherence video only 70% of the dots moved in one direction and the remaining 30% moved randomly (please see supplementary video files: Video S1–S4). Each dot had a lifetime of 1.6 s and a size of 0.6 cm×0.5 cm. The velocity was kept constant at 13°/s. The centre of rotation was always in between both eyes from the animal, as seen from above.

### Horizontal OKR Measurements

**Figure 1:**
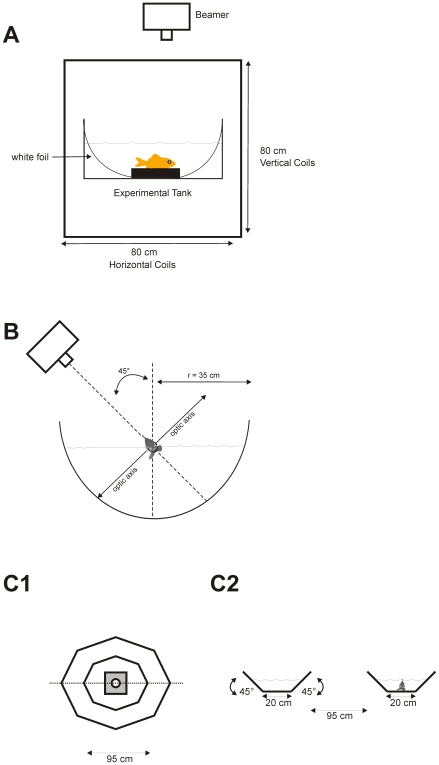
Schematic drawings of the experimental setup. **A** Section through the horizontal OKR setup. All experimental animals were placed in the middle of a circular tank which was placed in the middle of two horizontal and vertical coil pairs. Stimuli were projected from above, whereby the centre of rotation was always in between the eyes of the fish. To measure eye movements a search coil was attached to the right eye. **B** Frontal view of the electrophysiological setup. The goldfish was fixed in a plastic holder and artificially ventilated. The whole experimental setup was tilted 45° right side down so that the right eye was completely underneath the water surface for visual stimulation. A beamer produced a random dot pattern on the surface of the opaque hemisphere, whereby the axis of rotation was always in between the eyes of the fish. **C** OMR setup. Fish were allowed to swim freely within the ring shaped octagon tank, whereby the form of the tank forced the animals to swim around in a circular channel. C1 Ring shaped octagon tank seen from above (from the position of the video projector); video projector (light grey) was positioned in the center of the octagon tank. C2 Cross section through the tank along the dotted line in C1.

For horizontal eye movement recordings animals were fixed within a plastic fish holder and placed in the middle of pairs of horizontal and vertical coils (Fig. 1A). A search coil (1.2 mm diameter) was attached to the upper rim of the right eye with a tiny drop of acrylic glue and was held in place without damage or irritation of the cornea. Eye position signals were processed by lock-in-amplifiers (Princeton Applied Research, Model 128A), digitized, and stored on a computer hard disk (100 Hz). All experimental animals were placed in the middle of a circular tank (Ø 40 cm), which was covered with a skewed white foil. Stimuli were projected from above, whereby the centre of rotation was always in between the eyes of the fish (Fig. 1A). During optokinetic stimulation both eyes see either a clockwise (CW) or counterclockwise (CCW) horizontal rotation of the random dot stimulus.

Optokinetic eye movements were recorded for 30 s in each trial. After each experiment, the search coil used was detached, exactly repositioned in the magnetic field and calibrated with a protractor. The recorded calibration and eye position signal from the search coils were analyzed off-line with a custom made MATLAB program.

Ten out of nineteen fish were measured ten times and 9 fish were measured three times in each condition (0%–100% coherence) and direction (CW and CCW). For each condition the slope of ten slow phase eye movements was calculated to evaluate the gain of the slow phase eye movement.
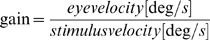



For each fish also an individual threshold was determined by judging from which coherence on slow phase eye movements or resetting saccades were visible in the eye traces.

### Electrophysiology

Before surgery animals were first anesthetized by immersion in a bath containing 0.1% 3-aminobenzoic acid ethyl esther (MS222). Anaesthesia was further supplemented locally with 2.5% lidocaine, before a craniotomy was performed to allow access to the left tectum opticum and pretectum. Immediately following surgery the animals were immobilized with Flaxedil (0.5–1 mg, i.m.) and transferred to a transparent recording hemisphere (diameter 70 cm), where they were artificially ventilated with cooled water (19°C). Single units were recorded with glass-coated tungsten microelectrodes (impedance 1–2.5 MΩ) in the left pretectum. For localizing direction-selective neurons in the APT the visual stimulus consisted of random light dots projected into the hemisphere by a planetarium projector centred above the fish's head (for further information of the experimental setup please see [24], [25]). Receptive field size and location of individual direction–selective neurons of the APT were tested qualitatively with single spots of light (diameter 4°–10°) projected by a hand lamp on the wall of the recording hemisphere. After identifying direction-selective neurons different coherence stimuli were projected by a beamer into the recording hemisphere (Fig. 1B). As for the behavioural experiments the perceived global motion is either a clockwise or counterclockwise horizontal rotation and the centre of rotation was always in between both eyes from the animal, as seen from above. Stimulus speed was kept constant at 13°/s, as former investigations from our laboratory showed that stimulation speeds around 10°/s are in the optimal velocity range of direction-selective neurons [23]. For all behavioural as well as electrophysiological experiments the same visual stimuli were used. All in all eleven different coherence stimuli, in steps of 10% were applied, ranging from 100% coherence to 0% coherence (Video S1–S4). Each trial consisted of a stationary phase (0–2000 ms), a rotation in one direction (2000 ms–5000 ms), another stationary phase (5000 ms–7000 ms) and a rotation in the opposite direction (7000 ms–10000 ms).

### Measurements of the Optomotor Response

In contrast to the OKR measurements animals were allowed to swim freely within a ring shaped octagon tank with 95 cm diameter and a water depth of about 15 cm (Fig. 1C). The 8 outer walls of the ring shaped octagon were tilted 45° outwards and the 8 inner walls 45° inwards such that a pattern projected from above covered the bottom (20 cm wide) as well as the tilted side walls. Thus the OMR would force the animal to swim around the circular channel.

Again different coherence videos were projected by a beamer; the centre of rotation was positioned to the centre of the ring shaped octagon channel. All stimulus parameters were the same as for the optokinetic measurements, except that here both eyes see the same stimulus direction (back to front or front to back). With e. g. back to front stimulus movement the fish perceived a motion like during drifting backwards. To compensate this the OMR should force the fish to swim forward. Animals were tested individually by inserting one by one into the experimental tank. Animals were allowed to accustom to the tank for 30 min in the dark. The presentation of stimuli with different coherence levels was randomized. Responses of the animals were videotaped for 2 min per stimulus direction and coherence level and analyzed off-line; the whole procedure was done four times with each fish.

To quantify the OMR the experimental tank was divided into four sectors. For each condition the number of sectors which the fish passed through in the direction of the stimulus (OMR), against the direction of the stimulus and the number of stationary phases were counted. To calculate the individual coherence threshold of each animal, the lowest coherence at which the number of responses in stimulus direction was significantly higher than the number of responses against the stimulus direction was determined. To assure the behavioral threshold of the OMR we analysed our data also by the use of a receiver operating characteristic (ROC).

### Data Analysis

To evaluate the OKR the median of all gains for each coherence step, direction and each fish was calculated. Median gains were plotted against the coherence level to visualize the behaviour of slow phase eye movements. We then compared with a t-test all obtained median gain values of one coherence level with the gain values of the subsequent lower coherence. A significant difference between both coherences indicates a decrease in OKR performance. This analysis shows the systematic dependence of the gain of optokinetic eye movements on the coherence level in random dot stimuli. We never observed smooth pursuit eye movements against the stimulus direction, so there was no possibility to apply a ROC analysis. Instead to determine the threshold at the population level we compared the number of trials in which we could observe a clear OKR independent of gain and number of slow phases. A sigmoid function was fitted to the data and threshold was set arbitrarily at 50% effective trials which is a conservative estimate.

Neuronal and OMR coherence thresholds were assessed with a neurometric function as described by Britten et al. [8]. In short, to determine the coherence thresholds of the recorded direction-selective neurons we calculated first for each coherence level the ROC for preferred and null direction, whereby each ROC is created by plotting the proportion of preferred direction trials on which the criterion level (firing rate from 0 to 250 Imp/s) is reached against null direction trials in which the same criterion is reached. In case of the OMR we calculated for each coherence level the ROC for swimming reactions in and against the stimulus direction. The response from one fish was in this case treated like one trial from the direction-selective neurons. Thus the OMR threshold mirrors the population response of all ten fish.

**Figure 2:**
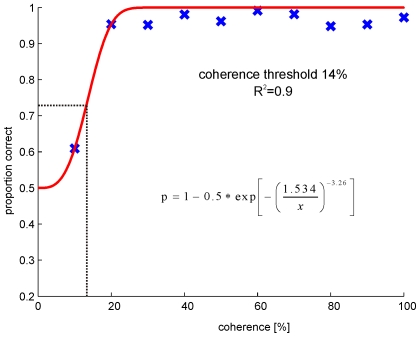
Neurometric function, which describes the sensitivity of one neuron to different coherence levels. The proportion of correct choices by the model is plotted against increasing coherence levels. The correlation level is the normalized area under the corresponding receiver operator curve (ROC). The red line corresponds to the fitted sigmoidal function. Threshold was estimated at the coherence level at which the model predicted 75% correct (dash dotted line). R^2^ corresponds to the coefficient of determination.

Afterwards the normalized area under the ROC of each coherence level was estimated and plotted against the coherence threshold (Fig. 2).These data were now fitted with a sigmoidal curve:
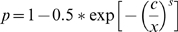
where c is the coherence level and s the slope of the function.

As threshold the coherence level at a proportion of 50% above chance (0.75 correct) was used. For a detailed description of threshold calculation please see Britten et al. [8].

## Results

### 1. Optokinetic Measurements

**Figure 3:**
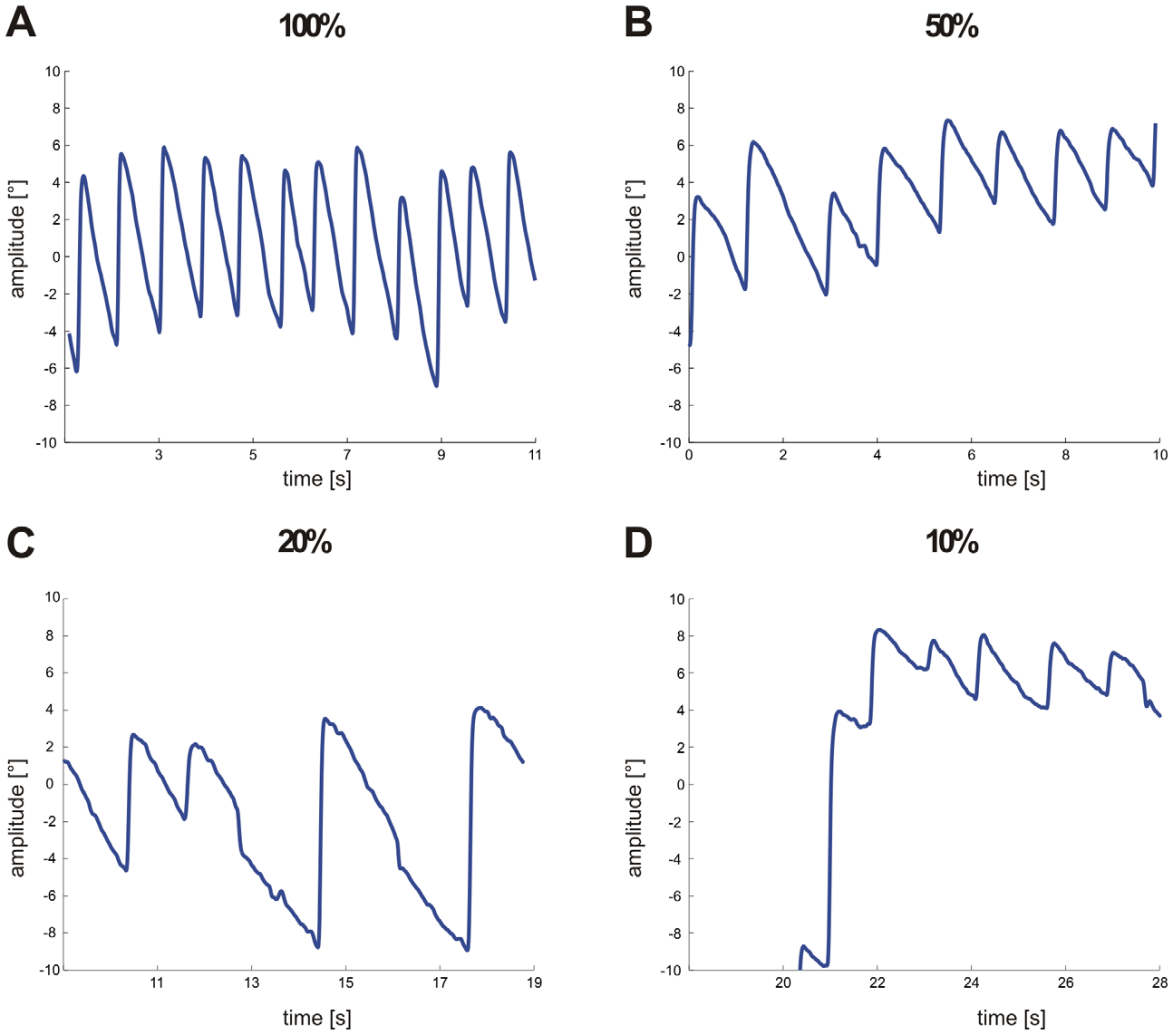
Typical examples of horizontal eye movement traces at different coherence levels. **A** 100% coherence. **B** 50% coherence. **C** 20% coherence. **D** 10% coherence.

Typical examples of slow phase eye movements during stimulation with different coherence levels are shown in Fig. 3. In all four conditions a regular nystagmus occurred. At 100% coherence (Fig. 3A) the median gain was 0.6 and the number of resetting saccades (n = 12) was largest (1/s) compared to all other conditions. With decreasing coherence levels gains declined significantly (t-test, p≤0.001) and at 50% coherence only a gain of 0.5 is reached. In this animal the decrease in gain resulted in both a drop in the number of resetting saccades (n = 9) and a drop in the amplitude of eye movements (Fig. 3B). Between 50% and 20% coherence no significant differences between gains (0.4) was evident (t-test, p≥0.356), only the number of resetting saccades decreased further (n = 4) compensated by an increase in amplitudes (Fig. 3C). In this animal slow phase eye movements during stimulation with 10% coherence were still visible (Fig. 3D) and even a gain of 0.2 is reached which was the highest at this level. In some animals the number of resetting saccades dropped, whereas in other animals the number of resetting saccades remained quite constant and only amplitudes of eye movements decreased with decreasing coherence levels.

**Figure 4:**
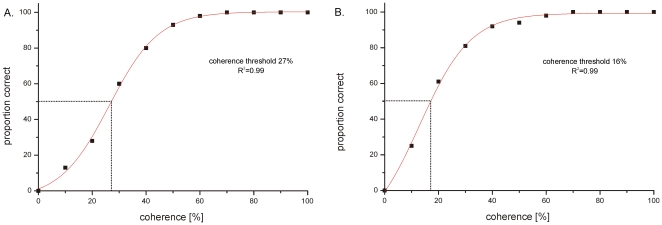
The percentage of trials in which a clear OKR was evident is plotted against the coherence level. A sigmoid function was fitted to the data and threshold was set arbitrarily at 50% effective trials. R^2^ corresponds to the coefficient of determination. **A** Clockwise direction. **B** Counterclockwise direction.

Slow phase eye movements were not evident in all animals at a coherence level of 10%, different individuals had varying thresholds for eliciting an OKR. In thirteen animals out of nineteen slow phase eye movements were already recognized during stimulation with 10% coherence, the remaining six animals had their threshold at 20% for eliciting an OKR at all. Slow phases could however not be elicited in every 30 s test trial especially at low coherence levels. We therefore determined the percentage of successful trials at each coherence level for each fish. When plotted against the coherence level and fitted with a sigmoid function a threshold set at 50% was determined. This threshold was taken because we used the same level for the OMR and the neuronal data. As figure 4 shows this conservative population threshold is reached at 27% in CW and at 16% in CCW direction.

Taken together our observation of individual animals and the population analysis show that even stimuli containing less than 20% coherently moving dots can trigger an OKR, although the likelihood to trigger an OKR decreases with decreasing coherences.

**Figure 5:**
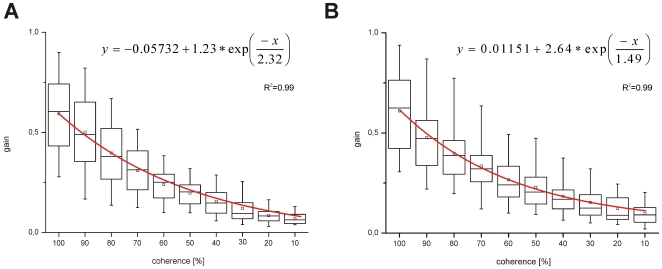
Median gains of optokinetic reactions in clockwise and counterclockwise direction over all animals. Data were only taken from those recordings in which clear slow phases were visible. The median gains were fitted with an exponential function (red line). R^2^ corresponds to the coefficient of determination. **A** Median gains in clockwise direction **B** Median gains in counterclockwise direction.

All nineteen animals showed a robust OKR in CW (median = 0.6) and CCW (median = 0.62) direction during the presentation of a 100% coherence stimulus at a velocity of 13°/s (Fig. 5A, B).Measured gains are comparable to gains which are reached with a planetarium projector or with a vertical black and white striped optokinetic drum [28]; [29]. When decreasing coherence by 10% steps gains significantly decreased (t-test, p≤0.001), independent from the presented direction. Gains declined in an exponential way and the lowest median gains of 0.08 and 0.09 in CW and CCW direction occurred with 10% coherence stimulation (Fig. 5A, B). Except for 40%, 30% and 20% (t-test, p<0.05) coherence we did not observe significant differences between gains in CW and CCW direction (t-test, p>0.05). On an individual basis, it becomes clear that observed asymmetries are not due to a systematic effect in favour of one direction, i.e.: in some fish gains for CCW directions were higher than for CW directions, whereas in other fish gains for CW direction were higher. In one animal asymmetries in both directions were recognized at different coherences.

### 2. Electrophysiology

**Figure 6:**
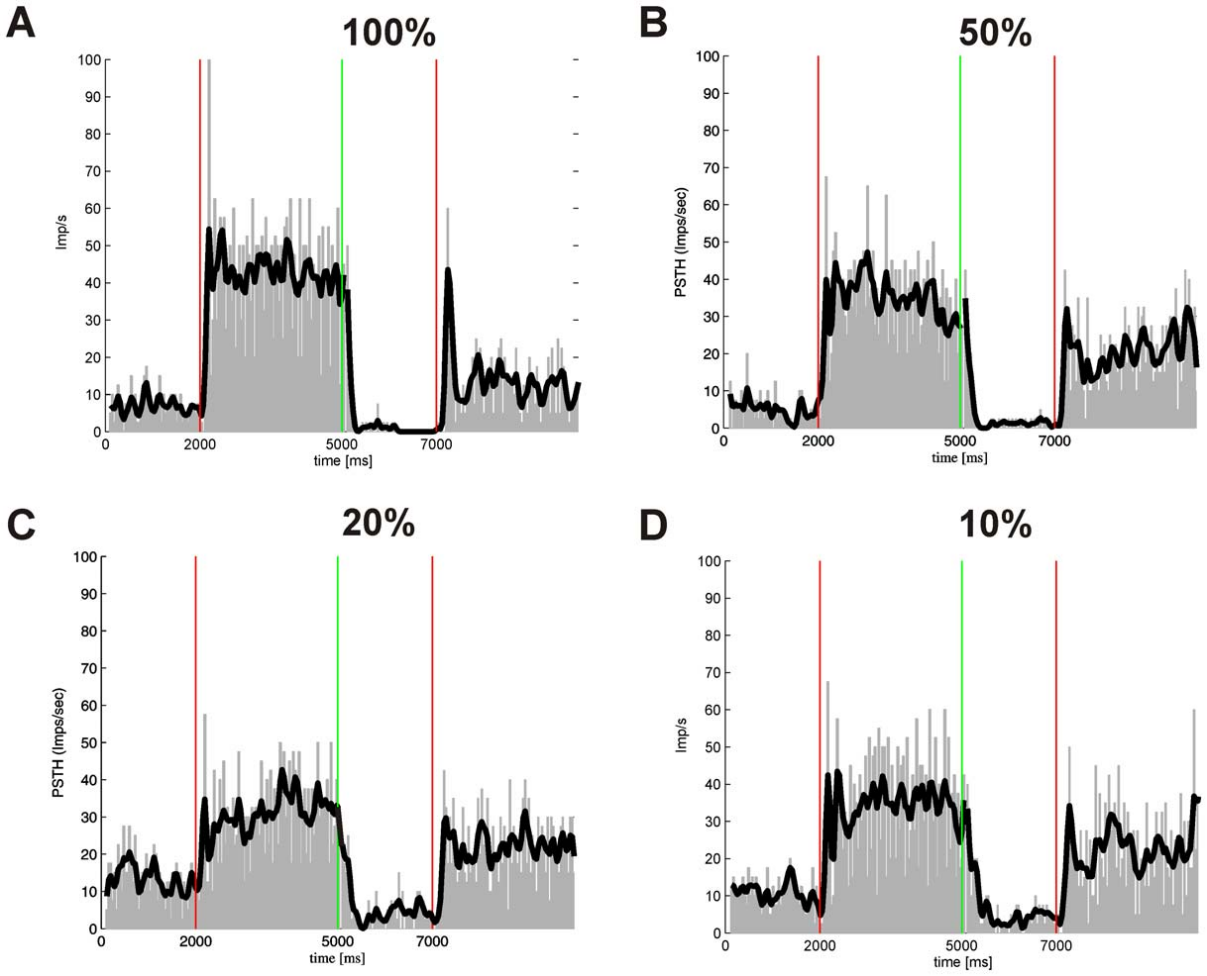
Typical example of a peristimulus time histograms of a direction-selective neuron in the pretectal area stimulated with different coherence levels. 0–2000 ms and 5000–7000 ms presentation of a stationary random dot stimulus, 2000–5000 ms stimulation in naso-temporal direction, 7000–10.000 ms stimulation in temporo-nasal direction. The beginning of the moving stimulus is marked by a vertical red line, whereas the green line marks the beginning of the stationary presentation of the random dot pattern. Black line corresponds to a Gaussian fitting of the spike train. **A** 100% coherence. **B** 50% coherence. **C** 20% coherence. **D** 10% coherence.

All in all thirty-seven direction-selective neurons with typical large receptive fields were recorded and tested with all coherence levels. Twenty-two of them had a stronger response to temporo-nasal and the remaining fifteen to naso-temporal stimulus direction as seen by the eye contralateral to the recording site. Since we only used horizontally moving stimuli we could not determine the exact preferred direction which could have been in any direction [23], [24], [25]. Nevertheless all neurons enhanced firing tonically during stimulation in one of the horizontal directions and were spontaneously active during presentation of the stationary random dot pattern. Figure 6 shows a typical example of a direction-selective neuron and its responses to stimuli with different coherence levels (100%, 50%, 20%, and 10%). In this neuron the firing rate in the preferred direction is rather independent of the coherence level, whereas the firing rate in the null direction increases with lower coherence probably due to weaker inhibition in the null direction with lower coherence. In other neurons the response in the preferred direction gets weaker with lower coherence and the response in the null direction remains rather constant.

**Figure 7:**
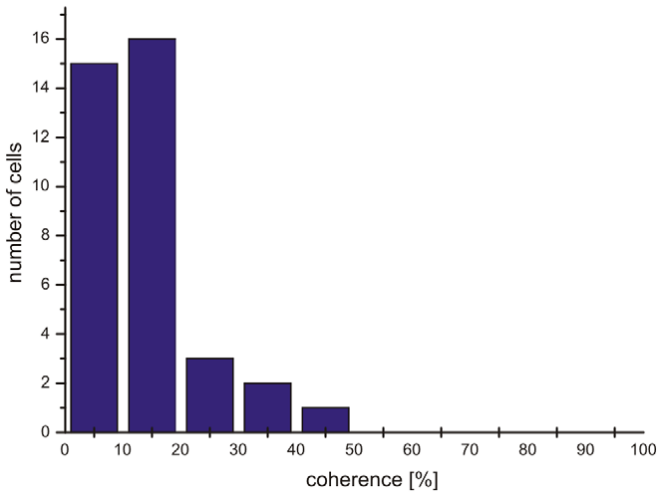
Frequency histogram of measured neuronal thresholds.

For each neuron a specific coherence threshold was assigned by a neurometric function which takes both the firing rate in the preferred and in the null direction into consideration (see materials and methods). The distribution of neuronal thresholds for all recorded neurons is given in Fig. 7. Forty-one percent of all recorded neurons had coherence thresholds of 10% or even lower, forty-three percent had thresholds between 10% and 20%, sixteen percent had thresholds between 20% and 50% coherence. Clearly, the majority of neurons (84 percent) had neuronal thresholds of less than 20% coherence matching the behavioural thresholds very well.

### 3. Optomotor Reaction

**Figure 8:**
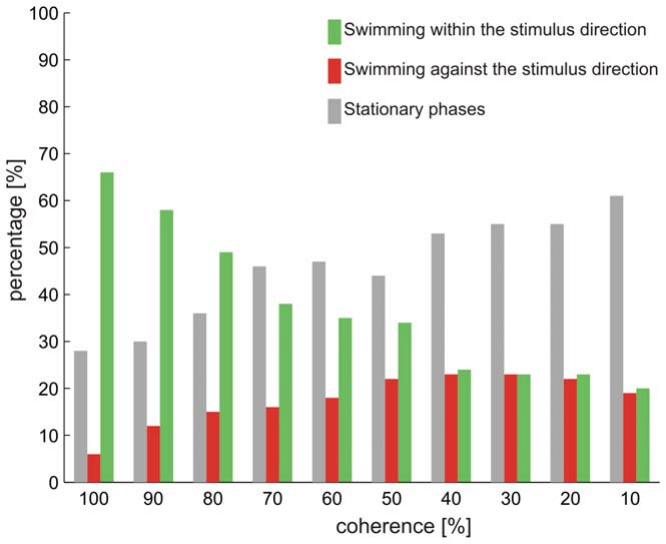
Example of the optomotor responses of one individual fish to stimuli with different coherence levels. Green bar: Swimming within the stimulus direction; red bar: Swimming against the stimulus direction; gray bar: Stationary phases.

A threshold for the optomotor response was determined in ten individuals. The stimulus with the lowest coherence at which animals showed significantly (t-test) more responses in than against the stimulus direction was ascertained as individual threshold. Figure 8 shows the responses of one animal to stimulation with different coherence levels. During the presentation of high coherence stimuli this animal exhibited a robust OMR, i.e. swimming in the stimulus direction. With decreasing coherence of the stimuli swim reactions against the stimulus direction increased, until at a level of 40% coherence, both response types were equally present. The amount of stationary phases increased also with increasing incoherence and forms the largest part of the response already at 60% coherence. Hence in this animal an individual coherence threshold of 50% was taken.

Nine out of ten animals had a coherence threshold between 40 and 50% and only in one animal a stimulus with 40% coherence was able to elicit an optomotor response.

**Figure 9:**
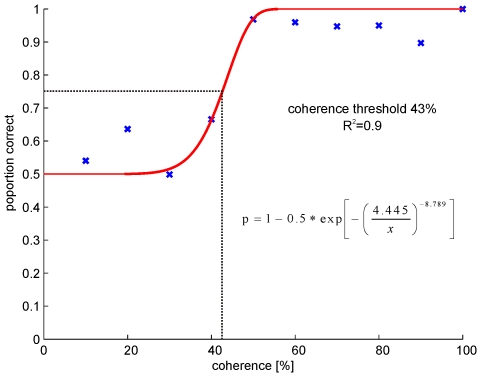
Psychometric function, which describes the sensitivity of the OMR to different coherence levels. The proportion of correct choices by the model is plotted against increasing coherence levels. The correlation level is the normalized area under the corresponding receiver operator curve (ROC). The red line corresponds to the fitted sigmoidal function. Threshold was estimated at the coherence level at which the model predicted 75% correct (dash dotted line). R^2^ corresponds to the coefficient of determination.

To approve that the behavioral threshold of the OMR is not influenced by noise or by our sample size, we used in addition the same data analysis as for the neuronal data. The ROC analysis results in a behavioral threshold of 43% and confirms the actual thresholds assessed in individual fish (Fig. 9).

## Discussion

Our objectives were to examine the coherence thresholds of the optokinetic response, the optomotor response and to determine neuronal thresholds of visual direction selective neurons in the pretectal area of goldfish. We find astonishing low thresholds for the optokinetic reaction and underlying neuronal circuits. In contrast to the optokinetic reaction (10% to 20% in individual cases; 16 to 27% on the population level) and visual direction-selective neurons (<20%) is the coherence threshold for the optomotor reaction is about 2 to 4 times higher (43%).

### Optokinetic Reaction

Results which were obtained with our 100% coherence random dot stimuli are by all means comparable to former studies. Already Dieringer [28] and Easter [29] showed that gains ranged from 0.4 to 0.68 during binocular stimulation in the goldfish and our mean gains obtained by stimulation with 100% coherence are within this interval. Even gain values not very close to unity are sufficient to improve vision, as image drifts up to several degrees per second are tolerated by the visual system and do not lead to blurred vision [30]. All in all this clearly shows that the stimulus used in our study is highly effective in triggering an OKR.

On a population level we observed only for some of the tested coherences levels a significant difference between gains in CW and CCW direction. Also other studies revealed slight asymmetries in the OKR of goldfish during binocular viewing conditions [29], [31], but the underlying mechanisms for this asymmetry are not yet clarified. Described asymmetries have no effects on OKR coherence thresholds, as median gains for both directions were always above thresholds responses. Furthermore at threshold stimulation no significant difference (t-test, p = 0.128) between gains in CW and CCW direction were evident.

As expected gains decreased with decreasing coherence of the presented stimulus, but the thresholds reached in our study are amazingly low compared to studies with other species [7], [8] except primates [9]. However, none of the other studies dealt with coherence thresholds of the optokinetic system, but instead investigated the perception of global motion, i.e. in all other studies the animals had to decide which direction within the random dots they perceived in a forced choice paradigm. It has to be determined if the perception of a certain direction can really be equated with the presence of an OKR or OMR.

Under normal conditions optokinetic stimulation always leads to slow phase eye movements following the direction of the stimulus. The animal can “decide” to follow or not to follow, but it cannot produce pursuit eye movements against the stimulus direction or when the stimulus is stationary. We never observed slow phases directed against the moving dots even at only 10% coherence. If slow phases occurred at all they would follow the direction of the coherently moving dots. If we assume that the presence of an OKR to low coherence stimuli is compatible with the threshold for the perception of the enclosed global motion our data can be compared with other studies. Of the species studied so far only humans and monkeys have better capabilities to detect global motion with a coherence threshold of 5% [7], [8]. Admittedly, our thresholds of 10%–20% might even be an overestimation of the real threshold for an optokinetic reaction, as we have not used coherence stimuli with less than 10% coherence. If we consider a median gain of 0.1 as the oculomotor threshold response some animals might actually be able to respond with a horizontal OKN to stimuli which contain less than 10% coherently moving dots (Fig. 3 and 5). Other species like pigeons and ferrets do not reach such low thresholds as the goldfish but have thresholds which are 2 to 4 times higher [7], [9].

### Global Motion Detection in Direction-Selective Neurons of the Optokinetic System

At least a coherence of 10% was necessary to elicit clear direction-selective responses in neurons of the APT. Up to date no other studies have dealt with global motion capabilities of the optokinetic system and our studies showed for the first time, which signal to noise ratio is needed by neurons of the APT to detect global motion. One study by Britten et al. [8] investigated coherence thresholds of visual direction selective neurons in MT of primates. Neurons in MT are involved in the analysis of visual motion perception. MT neurons had thresholds around 5% coherence and these thresholds correlated well with the observed discrimination performance of the monkeys. As MT cells provide a major input to the NOT and thus to the key structure driving OKR in monkeys [32] we believe that OKR in monkeys and man should also have a threshold near 5% coherence in random dot stimuli. This would further support the notion that the presence of an OKR is equal to the perception of global motion.

### Optomotor Response

Thresholds for the optomotor response were about 2 to 4 times higher than for the OKR. With a 100% coherence random dot stimulus OMR could reliably be triggered. Thus our design of an OMR stimulus seems adequate and therefore it is still highly astonishing that the actual threshold for the optomotor reaction lies at coherence levels of more than 40%.

Possibly the readiness of the fish to move the whole body during OMR is much lower than to move the eyes. In addition, real drifting in water will always generate a strong signal via the lateral line sensors which may be critical to trigger compensatory body movements. Another explanation for different thresholds of the OMR and OKR might be an imperfect read out of neuronal responses by the OMR system. The APT neurons respond well to optic flow generated by rotations. But APT neurons cannot differentiate between rotation and translation as their visual input is only mediated by the contralateral retina, i.e. occurring retinal slip during horizontal rotation or forward translation are more or less the same for monocular receptive fields. We do not know whether information from these neurons can be compiled to derive information about translational optic flow to trigger the OMR. But as long as the neuronal substrate for the OMR has not been analysed this remains hypothetical.

Due to the quite different threshold of the OMR compared to thresholds of direction-selective neurons and the OKR we presume that direction-selective neurons of the goldfish APT are not directly responsible for the OMR. Former lesion studies of the tectum opticum indicated an involvement of this structure in the OMR [14]. It seems likely that the OMR is not only mediated by the superficial visual layers of the tectum opticum [15], but rather by the intermediate and deeper layers of the tectum opticum. Various studies have shown the involvement of intermediate and deeper layers of the tectum opticum in the execution of eye, head and body movements [33], [34], [35], [36], [37], [38], [39], [40], [41]. These premotor structures might also be the underlying neuronal substrate of the OMR. As in fish there are no connections from direction selective neurons of the APT to the tectum opticum even an indirect involvement of APT neurons on the OMR is unlikely. At least it seems, as if there are two separated pathway, one mediating the OKR and the other mediating the OMR.

Studies, which investigate the coherence threshold of tectal direction-selective neurons and further lesion studies, are needed to clarify which is indeed the neuronal substrate for the OMR.

### Conclusion

Our study showed for the first time thresholds for global motion detection in a fish. The thresholds found in the optokinetic system, i.e. neuronal and behavioural threshold are unexpectedly low and come even close to perception thresholds of monkeys and humans. One of the possible explanations for differing thresholds for the OKR and OMR is that the OMR is not mediated by the optokinetic system, but rather by other motion detection systems.

## Supporting Information

Video S1 Example of a 100% coherence random dot stimulus in clockwise direction.
(1.21 MB MPG)

Video S2 Example of a 70% coherence random dot stimulus in clockwise direction.
(1.26 MB MPG)

Video S3 Example of a 10% coherence random dot stimulus in clockwise direction.
(1.29 MB MPG)

Video S4 Example of a 0% coherence random dot stimulus.
(1.29 MB MPG)
